# Clinical Presentation and Outcomes among Children with Sepsis Presenting to a Public Tertiary Hospital in Tanzania

**DOI:** 10.3389/fped.2017.00278

**Published:** 2017-12-22

**Authors:** Teresa Bleakly Kortz, Hendry R. Sawe, Brittany Murray, Wayne Enanoria, Michael Anthony Matthay, Teri Reynolds

**Affiliations:** ^1^Division of Critical Care, Department of Pediatrics, University of California, San Francisco, San Francisco, CA, United States; ^2^Institute for Global Health Sciences, University of California, San Francisco, San Francisco, CA, United States; ^3^Department of Emergency Medicine, Muhimbili University of Health and Allied Sciences, Dar es Salaam, Tanzania; ^4^Division of Pediatric Emergency Medicine, Department of Pediatrics, Emory University School of Medicine, Atlanta, GA, United States; ^5^Department of Epidemiology and Biostatistics, University of California, San Francisco, San Francisco, CA, United States; ^6^Division of Pulmonary and Critical Care, Department of Medicine, University of California, San Francisco, San Francisco, CA, United States; ^7^Department of Emergency Medicine, University of California, San Francisco, San Francisco, CA, United States

**Keywords:** global health, resource-limited, low-resource setting, pediatric critical care, pediatric emergency medicine, pediatric sepsis

## Abstract

**Background:**

Pediatric sepsis causes significant global morbidity and mortality and low- and middle-income countries (LMICs) bear the bulk of the burden. International sepsis guidelines may not be relevant in LMICs, especially in sub-Saharan Africa (SSA), due to resource constraints and population differences. There is a critical lack of pediatric sepsis data from SSA, without which accurate risk stratification tools and context-appropriate, evidence-based protocols cannot be developed. The study’s objectives were to characterize pediatric sepsis presentations, interventions, and outcomes in a public Emergency Medicine Department (EMD) in Tanzania.

**Methods:**

Prospective descriptive study of children (28 days to 14 years) with sepsis [suspected infection with ≥2 clinical systemic inflammatory response syndrome (SIRS) criteria] presenting to a tertiary EMD in Dar es Salaam, Tanzania (July 1 to September 30, 2016). Outcomes included: in-hospital mortality (primary), EMD mortality, and hospital length of stay. We report descriptive statistics using means and SDs, medians and interquartile ranges, and counts and percentages as appropriate. Predictive abilities of SIRS criteria, the Alert-Verbal-Painful-Unresponsive (AVPU) score and the Lambaréné Organ Dysfunction Score (LODS) for in-hospital, early and late mortality were tested.

**Results:**

Of the 2,232 children screened, 433 (19.4%) met inclusion criteria, and 405 were enrolled. There were 247 (61%) subjects referred from an outside facility. Approximately half (54.1%) received antibiotics in the EMD, and some form of microbiologic culture was collected in 35.8% (*n* = 145) of subjects. In-hospital and EMD mortality were 14.2 and 1.5%, respectively, median time to death was 3 days (IQR 1–6), and median length of stay was 6 days (IQR 1–12). SIRS criteria, the AVPU score, and the LODS had low positive (17–27.1, 33.3–43.9, 18.3–55.6%, respectively) and high negative predictive values (88.6–89.8, 86.5–91.2, 86.8–90.5%, respectively) for in-hospital mortality.

**Conclusion:**

This pediatric sepsis cohort had high and early in-hospital mortality. Current criteria and tested clinical scores were inadequate for risk-stratification and mortality prediction in this population and setting. Pediatric sepsis management must take into account the local patient population, etiologies of sepsis, healthcare system, and resource availability. Only through studies such as this that generate regional data in LMICs can accurate risk stratification tools and context-appropriate, evidence-based guidelines be developed.

## Introduction

Sepsis represents a spectrum of disease involving a systemic inflammatory response syndrome (SIRS) in the setting of infection, escalating in septic shock to cardiovascular and organ system dysfunction (1). It is the final common inflammatory pathway for most infectious disease-related deaths (2, 3) and incurs significant pediatric morbidity and mortality worldwide (1–9). Globally, there were 2.6 million deaths due to infectious diseases in children and adolescents in 2015, and 66% of these deaths occurred in sub-Saharan Africa (SSA) (10). A recent, global point prevalence study in children in pediatric intensive care units with severe sepsis from 128 sites in 26 countries, showed an aggregated in-hospital mortality rate of 25% (11). The identification and initial care of children with sepsis can significantly impact survival (12–14), and both delays in presentation (15) and delays in diagnosis have been shown to be risk factors for poor outcomes in low- and middle-income countries (LMICs) (14). The Surviving Sepsis Campaign’s international guidelines set standards for early identification, resuscitation, and protocol-based management, which have been shown to substantially impact outcomes (16). Much of the recommended sepsis management, especially regarding goal-directed interventions, is dependent on frequent or invasive monitoring that is not reliably available in limited resource settings and healthcare providers in LMICs often face resource constraints that limit capacity to implement these guidelines (13).

Beyond resource gaps that may limit the usability of international management guidelines, there is recent evidence suggesting that the clinical effectiveness of some interventions may be context-dependent. In 2011, Maitland et al., published results from a large, multicenter, randomized controlled trial: Fluid Expansion as Supportive Therapy (FEAST) (8). In this trial, African children with severe febrile illness who received fluid bolus resuscitation had a significantly higher relative risk (RR) of mortality compared to children who received only maintenance fluids (RR for any bolus vs. control, 1.45; 95% CI, 1.13–1.86; *P* = 0.003) (8). Current international guidelines recommend fluid bolus resuscitation for severe sepsis and septic shock (1, 16–20), but the unexpected results from the FEAST trial call into question whether practice guidelines primarily developed in high-income countries (HICs) are appropriate for LMICs. This may be especially true in malaria endemic regions given the increased possibility of shock secondary to severe anemia as compared to septic shock due to bacteremia (21, 22).

There is a critical lack of region-specific and relevant pediatric sepsis data, and few pediatric sepsis cohorts exist in SSA (7). While we recognize pediatric sepsis to be a global burden, we lack a robust understanding of how best to identify and manage sepsis in settings where triage and goal-directed therapy are challenging given the scarcity of invasive monitoring, laboratory tests, and medical personnel and where HIV, malaria, malnutrition, and limited access to care frequently complicate management. Tanzania is one such setting with a paucity of data on the profile and outcomes of children with sepsis. Thus, the objective of this prospective study was to characterize the clinical presentation, emergency interventions received, and outcomes among children with sepsis presenting to Muhimbili National Hospital (MNH) in Tanzania. The generation of region-specific data is needed to accurately risk stratify patients, develop appropriate protocols for early recognition, and implement evidence-based treatment protocols.

## Materials and Methods

This prospective descriptive study included pediatric patients presenting with sepsis to an urban, tertiary Emergency Medicine Department (EMD) at the National Referral Hospital in Dar es Salaam, Tanzania (MNH) between July 1 and September 30, 2016. The EMD is the receiving department for all acutely ill and injured patients, and is the only 24-h, full capacity public EMD in Tanzania. It is also the training site for the only emergency medicine residency in the country and cares for an average of 60,000 patients annually, and children comprise approximately 25% of the patient population. Pediatric sepsis is common at MNH; the EMD cares for an estimated 150–200 children with sepsis monthly (23, 24).

The EMD is equipped to provide plain radiographs, bedside ultrasound, continuous cardiorespiratory monitoring, low-flow oxygen, bag mask ventilation, intubation, mechanical ventilation, blood product transfusions, vasoactive medications, and resuscitation medications. MNH has a large general pediatrics ward, a high-dependency unit (HDU), and no dedicated pediatric intensive care unit. In general, the MNH ward can administer intermittent medications, intravenous fluids, low-flow oxygen, and blood products and measure vital signs every shift. The HDU is similar to the ward with a more favorable nursing ratio. Vasoactive infusions, intubation and mechanical ventilation are not routinely available for children outside of the EMD.

Children were included if they were between 28 days and 14 years of age and met criteria for sepsis, defined as having two or more clinical SIRS criteria (Box 1) with a suspected infection. At MNH, patients younger than 28 days present directly to the maternity and neonatal ward, and those over 14 years are triaged to adult rooms and admitted to the adult ward. SIRS criteria is defined per international pediatric sepsis consensus definitions (1), adapted for resource-limited settings (12). All pediatric patients presenting to the EMD were screened. Patients in cardiac arrest on presentation or with acute trauma were excluded. Written consent was obtained prior to enrollment. The primary outcome was in-hospital mortality. Secondary outcomes included EMD mortality and hospital length of stay.

Box 1 **Clinical SIRS Criteria for Resource-Limited Settings (12).**

Presence of ≥2 of the following 4 criteria with a suspected or proven infection:

- Abnormal temperature (axillary >38 or <36.0°C) documented or by history.
- Abnormal heart rate for age: tachycardia OR bradycardia (<1 year).
- Respiratory insufficiency: tachypnea for age, hypoxia (S_p_O_2_ <92%), NIPPV, OR mechanical ventilation.
- Ill-appearing, in distress, OR not responsive.

Patient data included: patient characteristics [age, sex, mid-upper arm circumference (MUAC), severity of illness]; patient comorbidities (HIV, malaria, TB, malnutrition); vital signs and SIRS criteria on admission; delay to care (onset of illness, facilities visited prior to MNH, duration of fever); socioeconomic status indicators (insurance status, parents’ education levels, parental literacy, number of children in the household, number of children under 5 in the household); interventions and therapies received in the EMD; relevant laboratory results; and outcomes. All diagnostic and therapeutic decisions were made at the discretion of the treating physician. Severe malnutrition was defined as a MUAC of ≤11.5 cm and severe anemia was defined as a hemoglobin ≤5 g/dL. Level of consciousness was assessed with the AVPU score (alert, responds to verbal stimuli, responds to pain, unresponsive) and severity of illness was measured with the 4-point Lambaréné Organ Dysfunction Score (LODS) (Box 2) (25). The LODS is a simple clinical prediction score for mortality validated in African children ≤15 years, including malaria endemic regions (25, 26). Early and late mortality were defined as death within 48 and ≥48 h of presentation, respectively. Microbiological investigations including cultures are not routinely performed on all patients, but results were recorded when available.

Box 2 **Lambaréné Organ Dysfunction Score (LODS) (2, 6, 25).**

Validated in African children ≤15 years with and without malaria. The score assigns one point for each of the following:

- Prostration—defined as ≥1 of the following, depending on age: inability to breastfeed, sit, stand, or walk unsupported.
- Coma—Blantyre Coma Score ≤2.
- Deep breathing.

Research personnel collected data from the electronic medical record, paper chart, care provider, and guardian. Study data were managed using REDCap electronic data capture tools (version 7.2.2) hosted at MNH (27). Data were deidentified prior to analysis. This study was carried out in accordance with the recommendations and approval of the Institutional Review Boards and Committees on Human Research at Muhimbili University of Health and Allied Sciences (Ref. No. 2016-03-30/AEC/Vol.X/201) and the University of California, San Francisco (IRB # 16-18977, Ref. No. 161295). We obtained written, informed consent from all guardians and assent from subjects when appropriate in accordance with the Declaration of Helsinki.

### Statistical Analysis

All data analysis and descriptive statistics were performed using Stata/MP (14.2), including means and SDs, medians and interquartile ranges, and counts and percentages as appropriate for the data type and distribution. We evaluated the ability of number of SIRS criteria, the AVPU score and the LODS to predict in-hospital mortality in this population. Scoring system discrimination was evaluated by comparing positive and negative predictive values.

## Results

**Figure 1:**
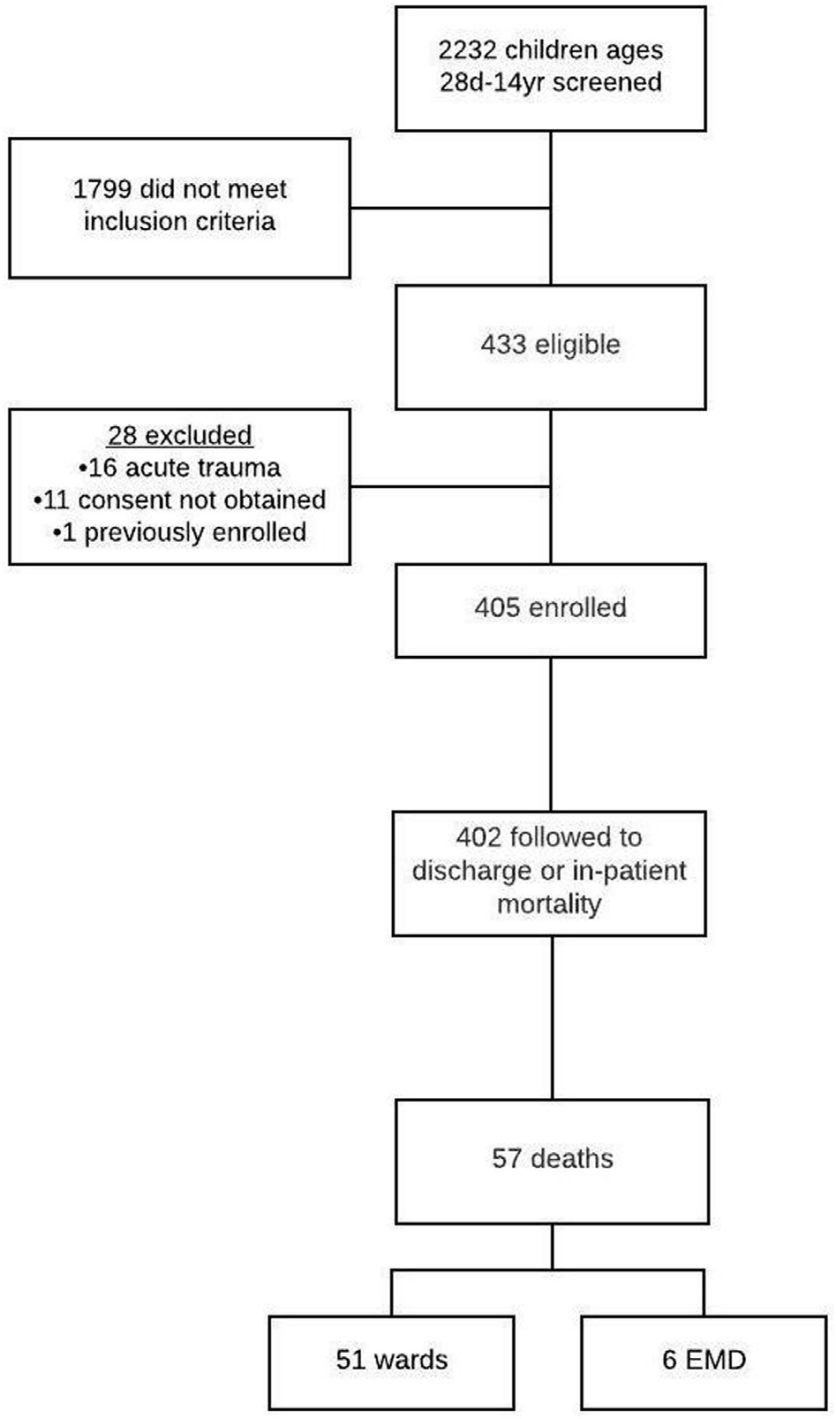
Patient screening, enrollment, and outcomes flowchart.

A total of 2,232 children presented to the EMD during the study period. There were 433 patients (19.4%) who had SIRS with a suspected infection, 28 children were excluded, and 405 subjects were successfully enrolled (Figure 1). Of those enrolled, 402 (99.3%) were followed to hospital discharge.

### Baseline Patient Characteristics

The median age of children with sepsis at MNH was 25 months (IQR 11–63 months), and 73.1% were under 5 years of age (*n* = 296) (Table 1). Severe malnutrition was present in 12.6% (*n* = 51) of children, 6.9% (*n* = 28) tested positive for malaria by rapid diagnostic test (RDT) or microscopy, 1.7% (*n* = 7) had confirmed (by RDT or antibody testing) or a known history of HIV, and 12.8% (*n* = 52) of subjects were tested for HIV during hospitalization. The mean hemoglobin was 8.3 g/dL (*n* = 305), and 16.1% (*n* = 49) of children presented with severe anemia. The mean venous blood gas pH among those tested (*n* = 184) was 7.36 (SD 0.14), while the mean lactate (*n* = 177) was 3.2 mmol/L (SD 3.5). The majority of children (61.7%, *n* = 250) presented with fever: of these, 36.4% (*n* = 91) had fever only by history and 63.6% (*n* = 159) had a documented fever on arrival. Almost 66% of patients (205/311) reported a fever duration greater than 48 hours and the mean fever duration was 8.7 days (SD 27.5). Over half of the patients (61%, *n* = 247) were referred to MNH from an outside hospital or clinic. Among referred patients, 47.5% (116/244) had been administered antibiotics before arrival to MNH. Up-to-date immunization status was reported in 97.3% (*n* = 394) of patients.

**Table 1:** Patient characteristics.

	Total (*N* = 405)
**Demographics**	
Age (months), median (IQR)	25 (11–63)
<2 years, *n* (%)	195 (48.2)
2–5 years, *n* (%)	101 (24.9)
>5 years, *n* (%)	109 (26.9)
Male sex, *n* (%)	236 (58.3)
MUAC, mean (SD)	14.7 (4.1)
≤11.5 cm, *n* (%)	51 (12.6)
Malaria positive, *n* (%)	28 (6.9)
HIV positive, *n* (%)	7 (1.7)
Fully immunized, *n* (%)	394 (97.3)
**Illness severity and level of consciousness**	
Lambaréné Organ Dysfunction Score	
LODS 0, *n* (%)	189 (46.7)
LODS 1, *n* (%)	144 (35.6)
LODS 2, *n* (%)	63 (15.6)
LODS 3, *n* (%)	9 (2.2)
AVPU score	
Alert, *n* (%)	331 (82.8)
Responds to verbal, *n* (%)	11 (2.8)
Responds to pain, *n* (%)	40 (10)
Unresponsive, *n* (%)	18 (4.5)
Missing	5 (1.3)
No. of SIRS criteria met, median (IQR)	3 (2–3)
2, *n* (%)	167 (41.2)
3, *n* (%)	166 (41)
4, *n* (%)	72 (17.8)
**Laboratory values**	
Hemoglobin (g/dL) (*n* = 305), mean (SD)	8.3 (3.0)
≤5 g/dL, *n* (%)	49/305 (16.1)
>10 g/dL, *n* (%)	85/305 (27.2)
WBC count ×10^3^ (*n* = 305), mean (SD)	16.6 (22.4)
Platelet count ×10^3^ (*N* = 304), mean (SD)	322 (201.6)
Venous blood gas pH (*n* = 184), mean (SD)	7.36 (0.14)
Bicarbonate (mEq/L) (*n* = 185), mean (SD)	21.3 (6.5)
Lactate (mmol/L) (*n* = 177), mean (SD)	3.2 (3.5)
Sodium (mEq/L) (*n* = 199), mean (SD)	132.5 (7.2)
Potassium (mEq/L) (*n* = 199), mean (SD)	4.1 (1.3)
Glucose (mmol/L) (*n* = 243), mean (SD)	6.6 (3.6)
**Prehospital course**	
Fever, *n* (%)	250 (61.7)
Documented, *n* (%)	159/250 (63.6)
By history, *n* (%)	91/250 (36.4)
Days of fever, mean (SD)	8.7 (27.5)
>2 days, *n* (%)	205/311 (65.9)
Hospital/clinic referral, *n* (%)	247 (61.0)
Antibiotics prearrival, *n* (%)	116/244 (47.5)
**Socioeconomic status indicators**	
Insured, *n* (%)	172 (42.5)
Exempt status, *n* (%)	131 (32.4)
Paternal literacy	372/388 (95.9)
Father’s highest level of education	
No formal school	12 (3)
Primary school	190 (46.9)
Secondary school	95 (23.5)
University or advanced degree	81 (20)
Unknown	27 (6.7)
Maternal literacy	372/403 (92.3)
Mother’s highest level of education	
No formal school	28 (6.9)
Primary school	228 (56.3)
Secondary school	78 (19.3)
University or advanced degree	62 (15.3)
Unknown	9 (2.2)
Number of children <18 in the household, median (IQR)	2 (1–3)
Number of children <5 in the household, median (IQR)	1 (1–2)
Electricity in the home	283/400 (70.8)
Improved water source, *n* (%)	403/423 (95.3)
Tap in the home, *n* (%)	96/403 (23.8)
Public tap, *n* (%)	214/403 (53.1)
Well, *n* (%)	93/403 (23.1)
Unimproved water source	20/423 (4.7)
Purchased water (bottled or truck)	9/20 (45.0)
Unprotected surface water	11/20 (55.0)
Toilet in the home	230 (56.8)

*IQR, interquartile range; MUAC, mid-upper arm circumference; LODS, Lambaréné Organ Dysfunction Score; AVPU, alert-verbal-pain-unresponsive score; SIRS, systemic inflammatory response syndrome; WBC, white blood cell*.

### Socioeconomic Status Indicators

Regarding insurance status, 42.5% (*n* = 172) of subjects were uninsured, though 76.2% (*n* = 131) of uninsured patients were categorically exempt from paying medical expenses by Tanzanian law based on age or disease status (Table 1). Paternal and maternal literacy rates were similar at 95.9 (*n* = 372) and 92.3% (*n* = 371), respectively, though more fathers completed secondary school or higher education than mothers (43.5 and 34.6%, *n* = 176 and 149, respectively). The median number of children younger than 18 years in the patient household was 2 (IQR 1–3). Ninety-five percent (*n* = 403) of families reported having regular access to an “improved” drinking water source, defined by the WHO/UNICEF Joint Monitoring Programme as a water source that is protected from sewage (28). Almost 71% (*n* = 283) of families had electricity, and 56.8% (*n* = 230) had toilets in the home.

### Illness Severity

On arrival, 82% (*n* = 331) of children were “alert” by the AVPU score. Using the LODS as a measure of illness severity, 46.7% (*n* = 189) of subjects had the lowest severity score of zero, while 17.8% (*n* = 72) had a score of two or more. The median number of documented SIRS criteria was 3 (IQR 2–3), with 41.2 (*n* = 167), 41.0 (*n* = 166), and 17.8% (*n* = 72) of subjects meeting 2, 3, and 4 criteria, respectively. Respiratory insufficiency was the most common SIRS criteria, occurring in 86.4% (*n* = 350) of patients, followed by abnormal temperature, abnormal heart rate, and abnormal appearance occurring in 70.9 (*n* = 287), 60 (*n* = 243), and 53.3% (*n* = 216) of patients, respectively (Table 2).

**Table 2:** Frequency of SIRS criteria for patients enrolled in the study.

SIRS criterion	Total (*N* = 405)
**Abnormal temperature, *n* (%)**	287 (70.9)
Temperature, mean (SD)	37.7 (1.0)
**Abnormal heart rate, *n* (%)**	243 (60)
Tachycardia, *n* (%)	235 (58.0)
Bradycardia, *n* (%)	8 (2.0)
**Respiratory insufficiency, *n* (%)**	350 (86.4)
Tachypnea, *n* (%)	330 (81.5)
Hypoxia, *n* (%)	76 (18.8)
S_p_O_2_, mean (SD)	95.5 (10.3)
**Abnormal appearance, *n* (%)**	216 (53.3)
Ill-appearing, *n* (%)	203 (50.1)
Distress, *n* (%)	21 (5.2)
Prostrate, *n* (%)	7(1.7)

*SIRS, systemic inflammatory response syndrome*.

### EMD Interventions

The most common EMD interventions included: antibiotic administration (54.1%, *n* = 219), fluid administration (38.8%, *n* = 157), oxygen therapy (24.5%, *n* = 99), chest radiograph (11.9%, *n* = 48), blood transfusion (11.2%, *n* = 45), and antimalarial medication administration (9.2%, *n* = 37) (Table 3). Three percent (*n* = 4) of pediatric sepsis patients had a blood culture drawn in the EMD, while 75.6% (*n* = 306) and 4.2% (*n* = 17) were tested with malaria and HIV RDTs, respectively. Few patients were intubated and received mechanical ventilation (1.2%, *n* = 5). While patients presenting in cardiac arrest were excluded, 1.2% (*n* = 5) of patients decompensated to require cardiopulmonary resuscitation (CPR) in the EMD.

**Table 3:** Interventions received in the EMD.

Intervention	*n* (%) (*N* = 405)
Fluid administration	157 (38.8)
Blood transfusion ordered	45 (11.1)
Blood initiated in EMD	25/45 (55.6)
Antibiotics received in the EMD	219 (54.1)
Antibiotics received prearrival, not in the EMD	40 (9.9)
Blood culture drawn in the EMD	4 (1.0)
HIV rapid diagnostic test	17 (4.2)
Malaria rapid diagnostic test	306 (75.6)
Vasoactive infusion	1 (0.2)
Hydrocortisone	1 (0.2)
Antimalarial medications	37 (9.1)
Oxygen	99 (24.4)
Chest radiograph	48 (11.9)
Ultrasound	12 (3.0)
Intubation and mechanical ventilation	5 (1.2)
CPR initiated	5 (1.2)
ROSC	1/5 (20)

*EMD, Emergency Medicine Department; CPR, cardiopulmonary resuscitation; ROSC, return of spontaneous circulation*.

Some form of microbiologic culture was collected during hospitalization in 35.8% (*n* = 145) of patients: blood, urine and cerebral spinal fluid (CSF) cultures were collected in 29.9, 14.8, and 4.0% of patients, respectively (Table 4). Of the cultures collected, 11.6, 10, and 0% of blood, urine and CSF were positive, respectively.

**Table 4:** Frequency of microbiologic tests performed and culture result positivity.

Microbiology	*n* (%)
Any microbiological culture performed	145/405 (35.8)
**Blood culture**	
Collected at any time during hospitalization	121/405 (29.9)
Culture positive	14/121 (11.6)
**Urine culture**	
Collected at any time during hospitalization	60/405 (14.8)
Culture positive	6/60 (10.0)
**CSF culture**	
Collected at any time during hospitalization	16/405 (4.0)
Culture positive	0/16 (0)

*CSF, cerebral spinal fluid*.

### Outcomes

**Figure 2:**
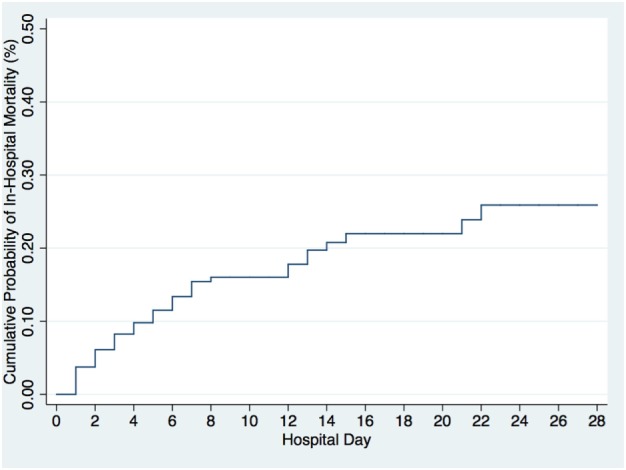
Cumulative probability of in-hospital mortality by hospital day.

**Figure 3:**
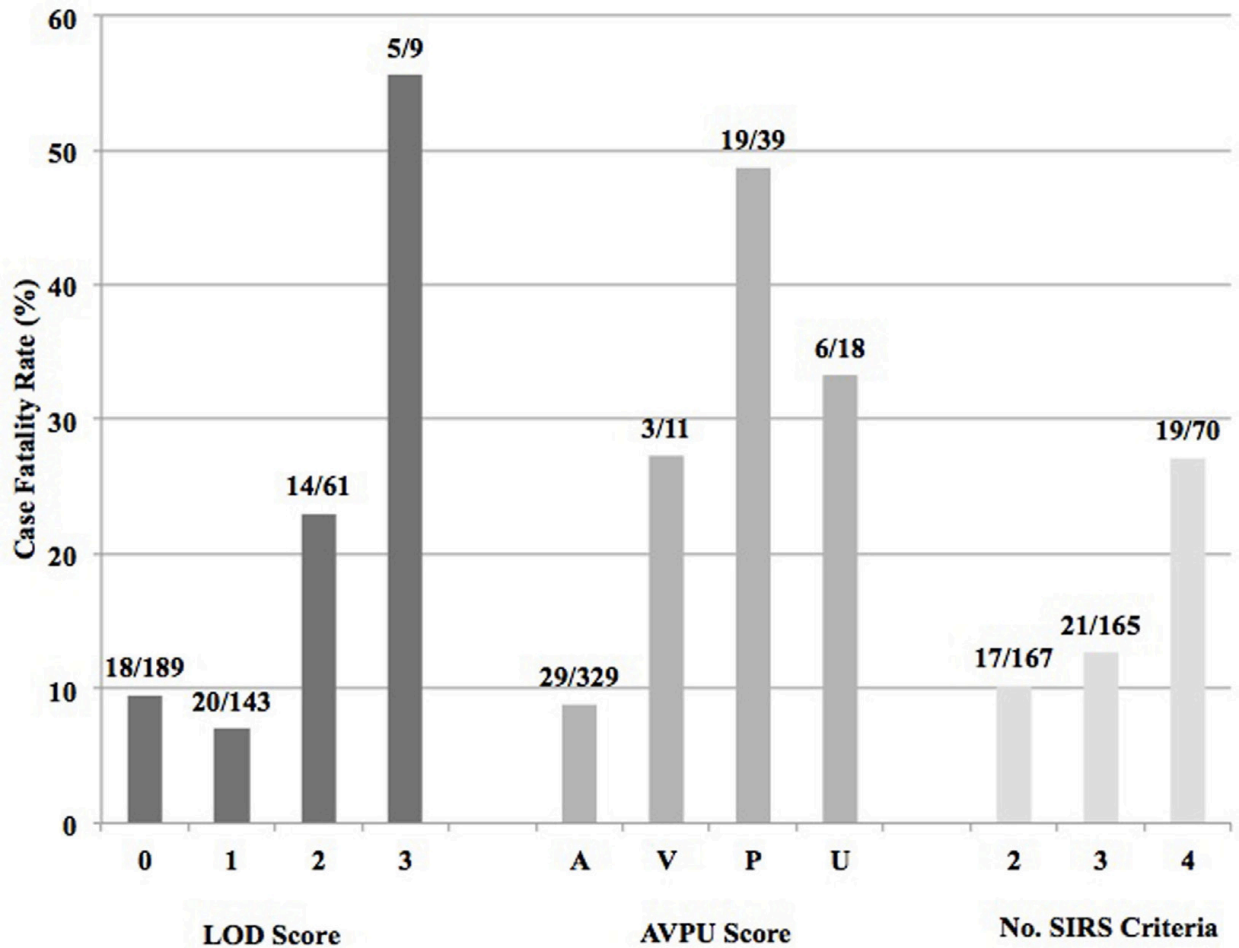
Case fatality rates by Lambaréné Organ Dysfunction Score, best response by the Alert-Verbal-Painful-Unresponsive (AVPU) score, and number of SIRS criteria present on admission.

Of the 402 patients followed to discharge, 57 patients died in the hospital, yielding an in-hospital mortality of 14.2% (Table 5). Six subjects died in the EMD, representing 10.5% (6/57) of the patients who died, and the overall in-ED mortality was 1.5% (6/402). The median time to death was 3 days (IQR 1–6 days) and 35.1% (*n* = 20) of the patients who died did so within 48-h of presentation. The cumulative probability of mortality rose sharply until hospital day 7 (15.4%) and then rose gradually until hospital day 22 when it plateaued at 25.9% (Figure 2). The median length of stay for all patients was 6 days (IQR 1–12 days). SIRS criteria and LODS were available on all 402 subjects, while AVPU scores and in-hospital outcomes were available on 98.6% (397/402) of subjects. All five of the patients with missing AVPU scores survived to discharge. In this population, case-fatality rates increased with rising LODS and number of SIRS criteria, but less so with worsening AVPU score (Figure 3). We explored early mortality with respect to interventions received in the EMD: 80% (*n* = 16) received a fluid bolus, two of who were malnourished, and 85% (*n* = 17) received antibiotics in the EMD (Table 6). All three scores—AVPU, LODS, and SIRS—had low positive predictive values, and higher negative predictive values for in-hospital, early and late mortality (Table 7). This was also true in patient subgroups with malnutrition and severe anemia.

**Table 5:** In-hospital outcomes for pediatric patients with sepsis.

Outcome	
EMD mortality, *n* (%)	6/402 (1.5)
In-hospital mortality, *n* (%)	57/402 (14.2)
Ward mortality, *n* (%)	51/57 (89.5)
EMD mortality	6/57 (10.5)
<48-h mortality, *n* (%)	20/57 (35.1)
≥48-h mortality, *n* (%)	37/57 (64.9)
Time to death (days), median (IQR)	3 (1–6)
Length of stay all subjects (days), median (IQR)	6 (1–12)

*IQR, interquartile range*.

**Table 6:** Early mortality due to sepsis and key EMD interventions.

	Early mortality (*N* = 20)
Fluid bolus in the EMD, *n* (%)	16 (80.0)
Malnourished, *n* (%)	2/11 (18.8)
Blood transfusion, *n* (%)	2 (10)
Severe anemia, *n* (%)	1/14 (7.1)
Antibiotics prearrival, *n* (%)	8/17 (34.5)
Antibiotics in the EMD, *n* (%)	17 (85.0)^a^
Antibiotics prearrival or EMD, *n* (%)	19 (95.0)
Antimalarial medication in the EMD, *n* (%)	4 (20.0)^b^
Malaria positive, *n* (%)	2 (10.0)

*^a^Six patients received antibiotics prearrival and in the EMD*.

*^b^Two patients tested negative and were treated*.

*EMD, Emergency Medicine Department*.

**Table 7:** Positive and negative predictive values for clinical scores (LODS, AVPU, SIRS criteria) predicting in-hospital (subgroups malnutrition and severe anemia), early and late mortality.

Severity of illness measure	Positive predictive value					Negative predictive value				

	Total (%)	Malnutrition (%)	Severe anemia (%)	Early mortality (%)	Late mortality (%)	Total (%)	Malnutrition (%)	Severe anemia (%)	Early mortality (%)	Late mortality (%)
**LODS**										
≥1	18.3	21.7	21.4	40.0	10.6	90.5	87.5	90.0	77.8	92.6
≥2	27.1	37.5	12.5	47.4	13.9	88.6	87.0	82.5	71.8	91.9
3	55.6	19.4	50.0	60.0	22.2	86.8	^a^	84.8	67.9	91.2
**AVPU score best response**										
Verbal or worse	41.2	42.9	16.7	39.3	24.6	91.2	87.5	83.3	70.0	94.0
Pain or worse	43.9	40.0	20.0	40.0	25.9	90.6	84.6	83.7	69.7	93.6
Unresponsive	33.3	^a^	^a^	50.0	16.7	86.5	78.6	82.6	67.3	91.1
**SIRS criteria**										
≥3	17.0	20.0	19.4	37.5	10.5	89.8	81.2	88.2	72.2	92.8
4	27.1	25.0	25.0	42.1	15.3	88.6	81.5	87.5	69.2	92.2

*^a^Too few subjects in a cell to calculate*.

*LODS, Lambaréné Organ Dysfunction Score; AVPU, alert-verbal-pain-unresponsive score; SIRS, systemic inflammatory response syndrome*.

## Discussion

This pediatric sepsis cohort had a high overall in-hospital mortality of 14.2%, substantially greater than a recent sepsis cohort in Uganda (5.4%) (29) and 48-h mortality in the FEAST trial (approximately 10%) (8). Almost 85% of deaths occurred within the first seven days of hospitalization, and over half of all deaths were within 72 h. Using SIRS criteria as a screening tool and requirement for study inclusion, the addition of the LODS, AVPU, or additional SIRS criteria were not useful in predicting in-hospital, early or late mortality in any of the tested subgroups, illustrating the difficulty of risk-stratification and mortality prediction in this patient population and setting.

The overwhelming majority of children who died did so on the ward and not in the EMD. Pediatric sepsis mortality occurring early in hospitalization, but outside of the EMD, has previously been observed in other SSA pediatric cohorts (8, 29). This may signify that despite acute stabilization, children are decompensating in the hospital, potentially due to poor monitoring, a lack of treatment resources, treatment failure, or the natural course of the disease; this study was not designed to assess the individual contribution of these factors and further investigation is needed.

Early recognition, resuscitation, and referral can be life saving in sepsis; yet, the majority of children had a fever duration over 48 h and were evaluated by providers at other health facilities prior to presentation at MNH, raising concerns about delays to definitive care. This, too, has been previously described; in South Africa, 40% of critically ill children were evaluated by >1 medical provider prior to referral to a tertiary care center, which resulted in delays in definitive care (30). Earlier recognition and targeted referral of high-risk patients by primary care providers could have a significant impact. It could be argued that the rate of antibiotic administration pre-arrival is a sign of provider recognition and treatment of sepsis, though additional exploration of referral patterns and therapies received in primary and secondary health facilities are required to address these aspects of care.

There are several limitations to this study. This cohort presented to an urban, tertiary center and may not reflect the general population in Tanzania. Parents in this cohort had a higher literacy rate (>90%) than previously reported for the general Tanzanian population (80.4%) (31) and 95% of families reported using an “improved” water source, significantly more than the 2015 national average of 55.6% (28), though there are legitimate concerns regarding tap water quality and safety (32, 33) in Tanzania. Given the inclusion criteria, this cohort was likely to be a sicker cohort than the general pediatric population; therefore, results cannot be extrapolated and applied to a general pediatric population. However, since a primary objective was to inform therapy guidelines and recommendations for the treatment of sepsis at a tertiary center, this was an ideal cohort to study.

We used SIRS criteria to identify patients with sepsis, and though these are the current, accepted criteria to identify pediatric sepsis, there has been much criticism regarding this operational definition (7, 29, 34). One criticism is that the definition of sepsis is highly context-dependent, especially in LMIC settings where diagnostic testing may be entirely unavailable or not available within a time frame relevant to guide acute care (7). In Uganda, researchers used SIRS criteria to identify sepsis in children with suspected infection; 86% met SIRS criteria, which captured 94% of all inpatient deaths, showing that SIRS criteria is highly sensitive for sepsis, but not specific, nor is it helpful in predicting mortality in this population and setting (29). The findings from this cohort study are consistent with the previous study results from Uganda.

Early mortality, as compared to late mortality, reflects EMD management and acute stabilization. In children who died within 48-h, 80% received a fluid bolus and 95% received antibiotics either pre-arrival or in the EMD. These findings could signify that guidelines were more closely followed in this subgroup, but failed to alter disease progression. Another possibility is that the intervention (i.e., fluid bolus) may potentially be contributing to early mortality as was seen in the FEAST trial (8). Further analyses are required to determine guideline compliance and the association of fluid bolus with mortality.

We found that pathogen identification with conventional microbiological techniques were low-yield and not frequently used, making it difficult to determine the etiology of sepsis. A malaria RDT was far more likely to be performed than a blood culture in children with sepsis. This may be due to several factors. First, a significant percentage of children received antibiotics prior to arrival to the EMD Second, the majority of blood cultures were drawn in the ward and not in the EMD and cultures were not routinely drawn prior to initiation of antibiotics. Third, blood culture collection supplies, specifically the bottles, may not always be readily available. Finally, some clinicians expressed general distrust of the microbiologic results and did not think the results changed management.

We also found that 36% (*N* = 146) of children did not receive antibiotics in the EMD or prior to arrival despite antibiotic administration being an essential component of international guidelines (1, 16, 18). The low rate of antibiotic administration may have resulted from limited drug availability, failure to identify sepsis, the need for urgent treatment, incomplete documentation of drug administration, or some combination of these factors. Further work is necessary to elucidate the underlying cause of low antibiotic usage in this setting. Depending on the cause, early antibiotic administration in the EMD for children with sepsis is a potential future quality improvement project.

Some data were extracted from the chart and may not have captured all interventions performed; however, the presence of research assistants actively observing actions in the EMD likely minimized this effect. Simultaneously, having research assistants physically present in the EMD may have influenced providers’ practices, producing an observer effect. The most likely results of such an effect would be increased provider awareness of sepsis and improved guideline compliance. We did not capture a metric for provider awareness, and guideline compliance in general was poor (not reported here), though antibiotic administration and blood culture collection are two indicators, which is consistent with prior pilot data from MNH.

In summary, pediatric sepsis management must take into account the local context; the unique patient population and its comorbidities, coinfections, and etiologies of sepsis; where and how the population accesses the healthcare system; and available resources including medical personnel and monitoring capabilities. Though there are many challenges to data collection in resource-limited settings—lack of research infrastructure, incomplete documentation, severe personnel shortages, inadequate funding, and technical difficulties performing follow-up—we have generated high-quality regional data that can be used to develop an accurate risk stratification tool and inform context-appropriate, evidence-based guidelines in this setting. This is the first, critical step towards improving pediatric sepsis outcomes in SSA.

## Ethics Statement

Research personnel collected data from the electronic medical record, paper chart, care provider, and guardian and then entered it into REDCap (version 7.2.2). Data were deidentified prior to analysis. This study was carried out in accordance with the recommendations and approval of the Institutional Review Boards and Committees on Human Research at Muhimbili University of Health and Allied Sciences (Ref. No. 2016-03-30/AEC/Vol.X/201) and the University of California, San Francisco (IRB # 16-18977, Ref. No. 161295). We obtained written, informed consent from all guardians and assent from subjects when appropriate in accordance with the Declaration of Helsinki.

## Author Contributions

Author contributions are as follows: conception and design of the work (TK, HS, BM, WE, and TR); data acquisition (TK, HS, and BM); data analysis (TK); data interpretation (TK, HS, BM, MM, and TR); first draft of the manuscript (TK); and manuscript revision and editing, final approval of the version to be published, and agreement to be accountable for all aspects of the work (TK, HS, BM, WE, MM, and TR).

## Conflict of Interest Statement

MM reports grants from Amgen and GlaxoSmithKline, personal fees from Roche-Genentec, Boerhinger-Ingelheim, Bayer, Quark Pharmaceuticals, Biogen, GlaxoSmithKline, Cerus Therapeutics, and Thesan Pharmaceuticals outside the submitted work. The other authors have no conflicts of interest to disclose; the research was conducted in the absence of any commercial or financial relationships that could be construed as a potential conflict of interest.
